# Mice prone to tinnitus after acoustic trauma show increased pre-exposure sensitivity to background noise

**DOI:** 10.3389/fnbeh.2023.1321277

**Published:** 2023-12-08

**Authors:** Natalia Rybalko, Štěpánka Suchánková, Zbyněk Bureš, Nataša Jovanović, Adolf Melichar, Oliver Profant, Rostislav Tureček

**Affiliations:** ^1^Department of Auditory Neuroscience, Institute of Experimental Medicine, Academy of Sciences of the Czech Republic, Prague, Czechia; ^2^Department of Otorhinolaryngology, Third Faculty of Medicine, University Hospital Královské Vinohrady, Charles University in Prague, Prague, Czechia; ^3^Second Faculty of Medicine, Charles University, Prague, Czechia

**Keywords:** noise exposure, hearing loss, tinnitus, acoustic startle reflex, background noise

## Abstract

Noise-induced tinnitus is generally associated with hearing impairment caused by traumatic acoustic overexposure. Previous studies in laboratory animals and human subjects, however, have observed differences in tinnitus susceptibility, even among individuals with similar hearing loss. The mechanisms underlying increased sensitivity or, conversely, resistance to tinnitus are still incompletely understood. Here, we used behavioral tests and ABR audiometry to compare the sound-evoked responses of mice that differed in the presence of noise-induced tinnitus. The aim was to find a specific pre-exposure neurophysiological marker that would predict the development of tinnitus after acoustic trauma. Noise-exposed mice were screened for tinnitus-like behavior with the GPIAS paradigm and subsequently divided into tinnitus (+T) and non-tinnitus (−T) groups. Both groups showed hearing loss after exposure, manifested by elevated audiometric thresholds along with reduced amplitudes and prolonged latencies of ABR waves. Prior to exposure, except for a slightly increased slope of growth function for ABR amplitudes in +T mice, the two groups did not show significant audiometric differences. Behavioral measures, such as the magnitude of the acoustic startle response and its inhibition by gap pre-pulse, were also similar before exposure in both groups. However, +T mice showed significantly increased suppression of the acoustic startle response in the presence of background noise of moderate intensity. Thus, increased modulation of startle by background sounds may represent a behavioral correlate of susceptibility to noise-induced tinnitus, and its measurement may form the basis of a simple non-invasive method for predicting tinnitus development in laboratory rodents.

## Introduction

Tinnitus is a phantom sound perception that reduces the quality of life of people, especially the elderly. Despite intensive research, its mechanisms remain only incompletely understood and there is essentially no effective treatment (Shore et al., 2016; Henton and Tzounopoulos, 2021). Tinnitus is usually triggered by hearing loss and very often by traumatic exposure to noise (Eggermont and Roberts, 2015). According to the generally accepted idea, cochlear damage caused by acoustic trauma leads to maladaptive changes in the auditory and non-auditory circuits and to the development of tinnitus (Roberts et al., 2010; Henry et al., 2014; Henton and Tzounopoulos, 2021). Remarkably, however, noise-induced hearing loss does not always lead to tinnitus (Konig et al., 2006), suggesting the existence of mechanisms underlying resistance to its development. Elucidating these could help to uncover the factors of hearing loss that contribute to tinnitus and suggest treatment options. In humans, differential susceptibility to tinnitus appears to be related to the severity of neuropsychiatric characteristics such as symptoms of depression, anxiety, stress or cognitive impairment (Trevis et al., 2018; Bhatt et al., 2022; Hamed et al., 2023). Research on tinnitus susceptibility using animal models, consistently showing that noise exposure leads to behavioral manifestations of phantom perception in only half of the subjects tested (Li et al., 2015; Mohrle et al., 2019; Fabrizio-Stover et al., 2022), then further suggested cellular and molecular mechanisms of resistance to tinnitus induction. These include plasticity of glutamatergic synapses in the cochlear nucleus, restoration of KCNQ2/3 potassium channel activity in principal dorsal cochlear nucleus neurons, potentiation of auditory brainstem responses along with expression of the immediate-early Arc/Arg3.1 gene, and increased GABAergic inhibition in the auditory cortex (Li et al., 2013; Ruttiger et al., 2013; Li et al., 2015; Heeringa et al., 2018; Miyakawa et al., 2019; Deng et al., 2020; Masri et al., 2021; also see Shore et al., 2016; Shore and Wu, 2019; Henton and Tzounopoulos, 2021 for reviews). On the other hand, less attention has been paid to investigating pre-exposure differences in animals with and without subsequent noise-induced tinnitus. Such research could help identify those predispositions in the central auditory system of naive animals that protect against tinnitus perception. This assumption is supported by the study of Ahlf et al. (2012), who found significantly higher pre-traumatic neuronal activity in the auditory system of gerbils resistant to noise-induced tinnitus and suggested that this activity could prevent the development of tinnitus through GABAergic inhibition. Similar studies require the use of techniques that allow for later behavioral detection of tinnitus and thus have limited ability to reveal details of the mechanisms involved, particularly at the cellular or subcellular level. It would therefore be useful to find a parameter that would allow animals to be sorted according to their sensitivity/resistance to noise-induced tinnitus and then to use their tissues for *ex vivo* and/or *in vitro* examination without prior induction of trauma. The aim of this study was to identify pre-exposure correlates of differential susceptibility to noise-induced tinnitus in mice using behavioral tests and ABR audiometry.

## Materials and methods

The care and use of animals were approved by the Ethics Committee of the Institute of Experimental Medicine, Academy of Sciences of the Czech Republic, and followed the guidelines of the EU Directive 2010/ 63/EU on the use of animals for scientific purposes.

### Animals

In this study, 61 two- to three-month-old C57BL/6 J mice of both sexes (27 males and 34 females) were used. The mice were housed in groups of 3 to 5 animals per cage under standard laboratory conditions, including a 12 h light–dark cycle, room temperature of 23°C, and unrestricted access to food and water.

### Noise exposure

To induce tinnitus, mice were exposed for 1 hour to 1/3 octave narrowband noise centered at 10 kHz and with an intensity of 116 dB SPL. During exposure, mice were anesthetized with a mixture of ketamine (Calypsol, 35 mg/kg) and xylazine (Xylapan, 6 mg/kg) and placed on a heated pad (37°C) in a custom-made soundproof chamber. Exposure noise was created using a white noise generator RFT 03004, processed using a set of filters RFT 01013 and a custom-made amplifier, and presented by a DE700 speaker (B&C Speakers, Italy) connected to a horn. The sound pressure level calibration was performed using a Brüel & Kjaer 4,939 1/4″ microphone and a B&K 2231 noise meter (Brüel & Kjaer, Denmark).

### Behavioral tests

Acoustic startle responses (ASRs) of mice were studied using the Acoustic Startle Response System (Habitest model E10-21, Coulbourn, Pennsylvania, United States) placed in a soundproof room. During testing, individual animals were confined in a small wire cage placed on a sensitive platform containing a load-cell transducer that converts pressure into voltages. The output electrical signals were acquired and processed using a TDT 3 system with an RP 2 real-time processor (Tucker Davis Technologies, Alachua, United States). Acoustic stimuli were generated by a TDT 3 system and presented through a 29TAF/W loudspeaker (SEAS, Norway) placed above the animal. The frequency response of the loudspeaker in the test rig was within ±9 dB at 1–32 kHz. The equipment was calibrated using a Brüel & Kjaer 4,939 1/4-inch microphone and a Brüel & Kjaer 2,231 sound meter (Brüel & Kjaer, Denmark). Stimulus generation, data collection and processing were controlled by Matlab software. Startle responses were analyzed within a 100 ms window beginning at stimulus onset and were measured as maximal peak-to-peak voltage amplitude.

**Figure 1:**
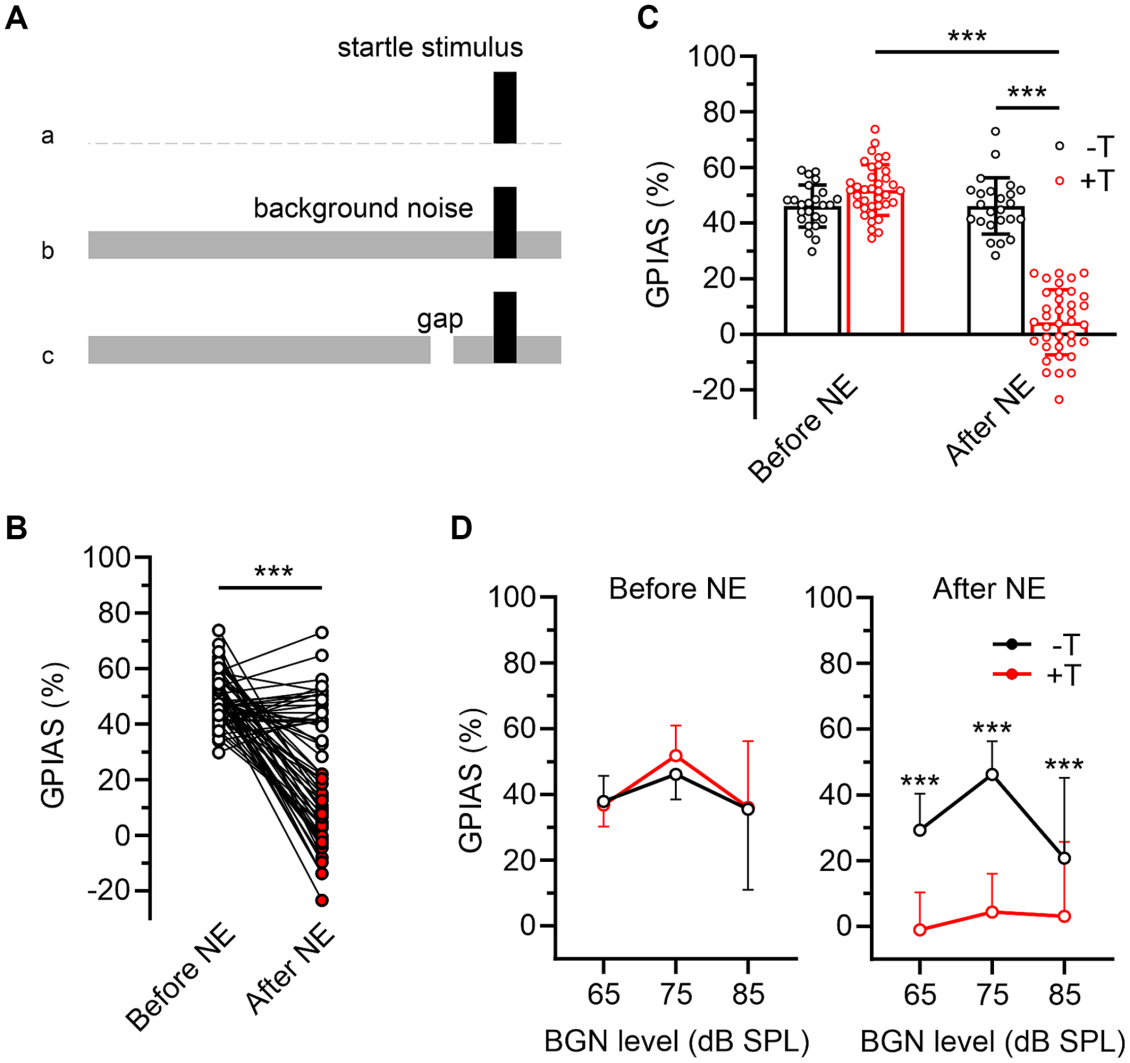
Two groups of mice differing in behavioral symptoms of noise-induced tinnitus. **(A)** Behavioral paradigms used to assess baseline raw ASR (a), ASR inhibition in the presence of BGN (b), and inhibition of ASR by gap pre-pulse in BGN (c). **(B)** Magnitudes of ASR inhibition by a gap embedded in 75 dB BGN (GPIAS) in 61 mice before and after noise exposure (NE). White and red filled symbols indicate post-exposure GPIAS values in mice in which there was (−T, *n* = 24) or was not (+T, *n* = 37) a significant difference between ASR_gap_ and ASR_no gap_ after NE (see Methods for calculation). **(C)** The bar graph compares the mean GPIAS values for −T and +T groups before and after NE (*p* = 0.060 and *p* < 0.001; two-way ANOVA with SMCT). Note that after NE, GPIAS did not change significantly in −T mice (*p* = 0.999) whereas it decreased dramatically in +T mice (*p* < 0.001; two-way RM ANOVA with SMCT). **(D)** Differences in pre- and post-exposure GPIAS between −T and +T mice are similar at three different BGN levels (***p < 0.001; mixed-effects model with SMCT).


Testing consisted of two identical sessions, conducted separately on two consecutive days. The aim of the first session was to adapt the animal to the test equipment and its results are not presented in this study. Each session began with a ten-minute habituation of the animal to the experimental setup, followed by 4 consecutive trains of 21 startle-eliciting stimuli at intervals randomly varying from 20 to 30 s. Intertrain intervals were 10 min, and responses to the first of 21 stimuli for each train were not included in further analysis. The sessions included three different types of behavioral paradigms (Figure 1A): (a) induction of ASR by the startle stimulus alone (white noise, 50 ms/110 dB SPL, rise/fall time 0.5 ms) without further modulation, (b) ASR inhibition by narrowband background noise (BGN, 1/3 octave band centered at 10 kHz, 65–85 dB SPL), and (c) inhibition of ASR by gap pre-pulse (50 ms, rise/fall time 3 ms, 100 ms time interval between onset of pre-pulse and startle stimulus) in the presence of BGN (GPIAS). The paradigm in (a) was applied during the first trains of the sessions, while the paradigms in (b) and (c) were applied during the second to fourth trains as 10 no gap and 10 gap trials presented in a random order in the presence of continuous BGN of 65, 75, 85 dB SPL intensity, different for each train. The raw ASR amplitude for each mouse was quantified as the average of 10 randomly selected voltage responses recorded during the first train of the session. ASR inhibition by BGN was quantified as follows:

$$\mathrm{Relative}\;ASR\;\mathrm{amplitude}\;\left[\%\right]=\left({ASR}_{BGN}/{ASR}_{\mathrm{no}\;BGN}\right)\times 100\text{,}$$

where ASR_BGN_ and ASR_no BGN_ represent the average raw ASR amplitudes obtained from 10 replicate measurements for trials with no gap in the presence of BGN and from measurements obtained during the first train in the absence of BGN (see above). ASR inhibition by gap-prepulse was quantified as follows:


$$\mathrm{GPIAS}\;\left[\%\right]=\left(1-\left[{ASR}_{\mathrm{gap}}/{ASR}_{\mathrm{no}\;\mathrm{gap}}\right]\right)\times 100\text{,}$$

where ASR_gap_ and ASR_no gap_ represent the average raw ASR amplitudes, each obtained from 10 repeated measurements during the same stimulus train in the presence of BGN, with and without gap pre-pulse. The significance of the difference between ASR_gap_ and ASR_no gap_ was further tested using unpaired Student’s *t*-test for each mouse. Mice that showed non-significant differences between ASR_gap_ and ASR_no gap_ at 75 dB BGN 2 weeks after acoustic trauma were assigned to the tinnitus group (+T), while mice showing any significant differences were assigned to the non-tinnitus group (−T).

### Audiometry

Auditory brainstem responses (ABRs) were obtained from ketamine/xylazine anesthetized mice using methods described previously (Willott, 2006; Rybalko et al., 2019). During recording, mice were placed on a heated pad in a soundproof anechoic room. A TDT System 3 Real-Time Processor RP 2.1 (Tucker Davis Technologies, FL, United States) was used to generate sound stimuli that were transmitted to the animal via a PMA 720 acoustic system (Denon, Japan) and a loudspeaker system consisting of a 140-15D ribbon tweeter (RAAL advanced loudspeakers, Serbia) and an Alpha 6A speaker (Eminence, United States) under free-field conditions. The frequency response of the system was flat (±3 dB) in the range from 1 to 50 kHz. Calibration of the equipment was performed using a Brüel & Kjaer 4,939 1/4-inch microphone and a B&K 2231 sound level meter (Brüel & Kjaer, Denmark).

ABRs were elicited by clicks of 0.1 ms duration and a series of tones of 2, 4, 8, 16, and 32 kHz and 5 ms duration, including 1 ms onset and offset ramps with a rate of 15/s. The level of both types of stimuli was set between 100 and 0 dB SPL and gradually decreased in steps of 5 or 10 dB. Evoked brainstem potentials were recorded using subcutaneous needle electrodes placed on the vertex (active electrode) and in the region of the neck muscles (reference electrode). Recorded signals were band-pass filtered (300 to 3,000 Hz) and averaged from 500 traces elicited by repetitive stimulation (TDT BioSig software). The ABR threshold was the minimum tone or click intensity that elicited a visually detectable ABR within the expected time window. The amplitude of ABR waves I and IV was measured from the negative peak to the positive peak in responses evoked by click stimuli. The amplitudes of ABR waves I and IV obtained at varying click levels were used to construct ABR growth functions. To determine the slope of the function, for each mouse, the region of the function starting 10 dB above threshold was fitted using linear regression. Absolute latencies were measured from the time point at which the sound stimulus reached the ear to the time of the positive peak of each wave.

### Statistical analysis

The datasets were analyzed using GraphPad Prism 9.5. All values are presented as the mean ± SD, the significance level is set to be <0.05. Two-sample Student’s *t*-tests and two-way ANOVA followed by Sidak’s multiple comparison test (SMCT) were used to assess differences in means, unless otherwise stated. The normality of the data was assessed using D’Agostino and Pearson’s test and the presence of outliers was checked using the ROUT method (Q = 0.05%). Correlations between variables were assessed using Pearson and Spearman correlation coefficients.

## Results

### Differential sensitivity of mice to tinnitus induction

To induce tinnitus, we exposed anesthetized mice (*n* = 61) to 1/3 octave narrow-band noise, centered at 10 kHz with intensity of 116 dB SPL for 1 hour (Rybalko et al., 2019). The presence of tinnitus was assessed by inhibition of ASR with a gap pre-pulse in BGN (Turner et al., 2006) (Figure 1A). The intensity of BGN was first set to 75 dB SPL which is well audible even after hearing loss induced by acoustic trauma (see later). Two weeks after exposure, mice showed a significant decrease in the magnitude of ASR inhibition by gap pre-pulse (GPIAS) from 49.6 ± 8.9% to 20.8 ± 23.4% (*p*<0.001, paired Student’s *t*-test) (Figure 1B). This was consistent with the presence of noise-induced tinnitus, which masked gap pre-pulse, thereby reducing its inhibitory effect on ASR. The simultaneous increase in the coefficient of variation of GPIAS values from 18.0 to 112.6% suggests heterogeneity in the effect of exposure on the development of tinnitus. To distinguish between mice with high and low severity of tinnitus symptoms, we divided the entire group of animals into those that exhibited either non-significant or any significant inhibition of ASR by the gap embedded in 75 dB BGN after exposure (Figure 1B). Significance of inhibition in each mouse was assessed by statistical comparison of the differences between mean raw ASRs calculated from repeatedly stimulated responses with and without a gap pre-pulse (Kraus et al., 2011) (see Methods). These two distinct groups are hereafter referred to as tinnitus (+T) and non-tinnitus (−T) (Figure 1C). Of note, pre- and post-exposure GPIAS values were not significantly different in males and females (before exposure: *p* = 0.961; after exposure: *p* = 0.979; SMCT, data from 27 males and 34 females) and in subsequent analyses, data from mice of both sexes were pooled together.

Despite the tendency of +T mice to have higher GPIAS values than −T mice before exposure, the differences did not reach statistical significance (Figure 1C). Accordingly, the correlation between pre- and post-exposure GPIAS values in the whole mouse population was not significant (Spearman *r* = −0.233, *p* = 0.071, *n* = 61). Furthermore, mice of both groups showed similar raw ASR amplitudes in the absence or presence of 75 dB BGN after exposure (for −T and + T mice, ASR_no BGN_ were 0.631 ± 0.244 V and 0.695 ± 0.353 V, *p* = 0.603, and ASR_no gap_ were 0.399 ± 0.167 V and 0.368 ± 0.247 V, *p* = 0.881, two-way RM ANOVA with SMCT, *n* = 24 and 37), and no significant correlation was observed between these amplitudes and postexposure GPIAS at 75 dB BGN (ASR_no BGN_: Spearman *r* = 0.009, *p* = 0.945, ASR_no gap_: Spearman *r* = 0.188, *p* = 0.147, *n* = 61). Finally, a 10 dB SPL shift in BGN level up or down did not alter the significance of differences in GPIAS between the two groups (Figure 1D). These observations indicate that noise induced GPIAS failure in +T mice did not depend on the baseline value of this index before exposure or on its parameters such as post-exposure ASR amplitude and BGN intensity. Thus, our results support that the +T and −T groups differ in the presence or intensity of tinnitus and are consistent with the previously described heterogeneous sensitivity of mice to noise-induced tinnitus (Li et al., 2015; Fabrizio-Stover et al., 2022).

### Auditory function in mice prior to tinnitus induction

**Figure 2:**
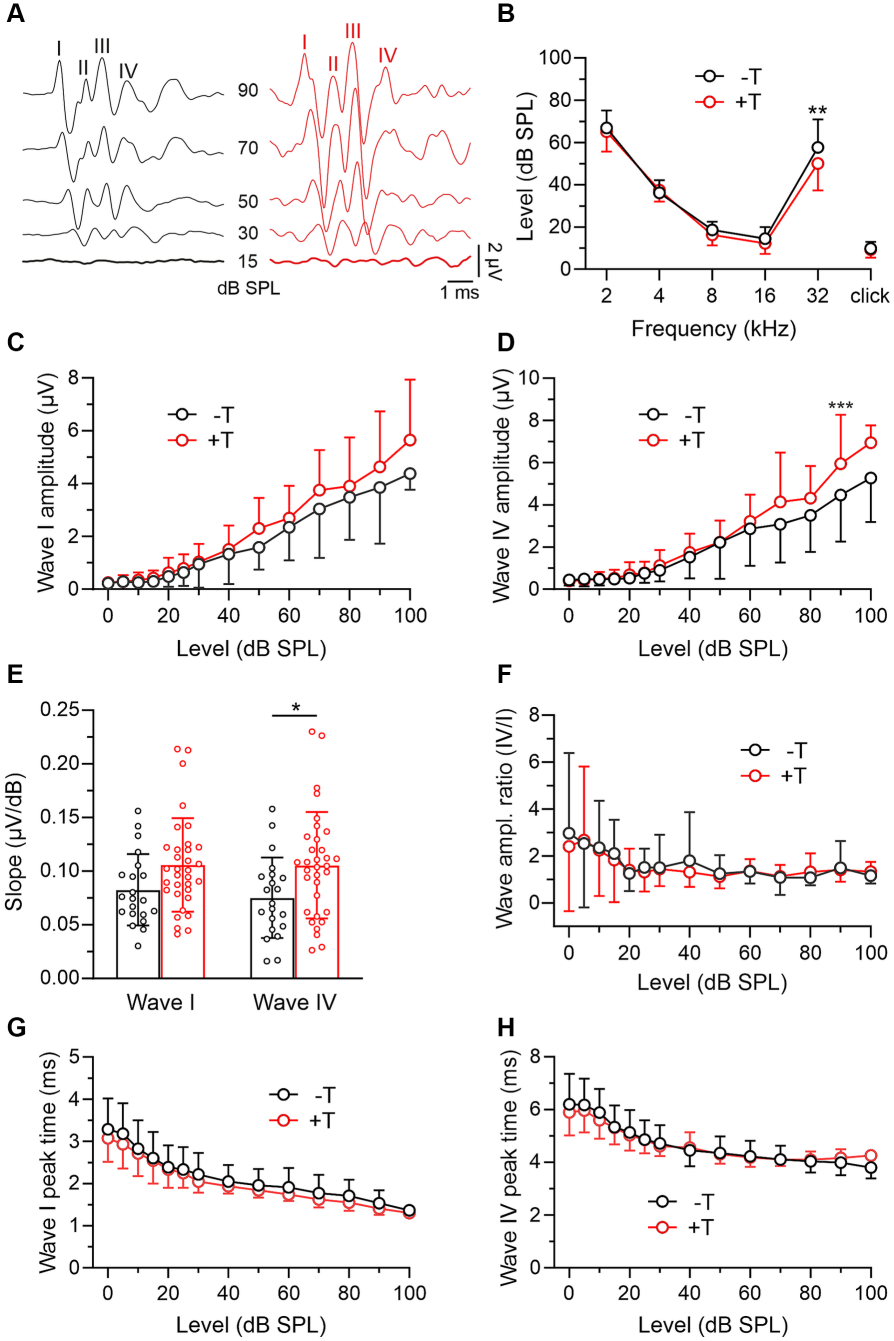
Comparison of ABRs in −T and +T mice before noise exposure. **(A)** Representative ABR recordings elicited by clicks of different intensities in mice from the −T and +T group before NE. Roman numerals indicate consecutive ABR waves. Responses evoked by threshold stimuli are shown in bold. **(B)** Thresholds of ABRs evoked by tones (2–32 kHz) or clicks in 24 mice from the −T group and 34 mice from the +T group. Except for those elicited by 32 kHz stimuli (*p* = 0.001), ABR thresholds were not significantly different in −T and +T mice (two-way ANOVA with SMCT). **(C,D)** Average amplitude-level functions for click-evoked ABR waves I and IV in 21 and 34 mice from the −T and +T groups, respectively. Growth functions for both waves were significantly different in −T and +T mice (*p* < 0.001, two-way ANOVA; ***p < 0.001, SMCT). **(E)** The bar graph compares the slope of the growth functions of ABR waves in −T (black) and +T (red) mice (*p < 0.05, two-way ANOVA with SMCT). **(F)** Similar ABR wave IV/I amplitude ratio in −T and +T mice (*p* = 0.547; two-way ANOVA with SMCT). **(G,H)** Dependence of latency of ABR waves I and IV on stimulation intensity in −T and +T mice. No significant difference was found between mouse groups at any click stimulus level (two-way ANOVA with SMCT).

In these experiments, we compared the auditory function in mice from the +T and −T groups before noise exposure. We first analyzed bilateral ABRs evoked by pure tones and click stimuli (Figure 2). The thresholds of ABRs showed the lowest values in the frequency range of 8 to 16 kHz, consistent with previously reported values (Zheng et al., 1999), and were not significantly different in +T and −T mice (Figure 2B). On the other hand, ABR wave amplitudes showed a slight increase at higher sound levels in +T mice (Figures 2C,D), resulting in a steeper slope of their input–output functions (Figure 2E). The increase was manifested by the amplitudes of both early and late ABR waves (I, IV), so that their ratio (IV/I), a measure of neural gain (Mohrle et al., 2019), was similar in both animal groups (Figure 2F). Peak latencies were not significantly different in +T and −T mice (Figures 2G,H). Thus, our findings show that mice that significantly vary in their susceptibility to noise-induced tinnitus do not show substantial differences in their ABRs.

**Figure 3:**
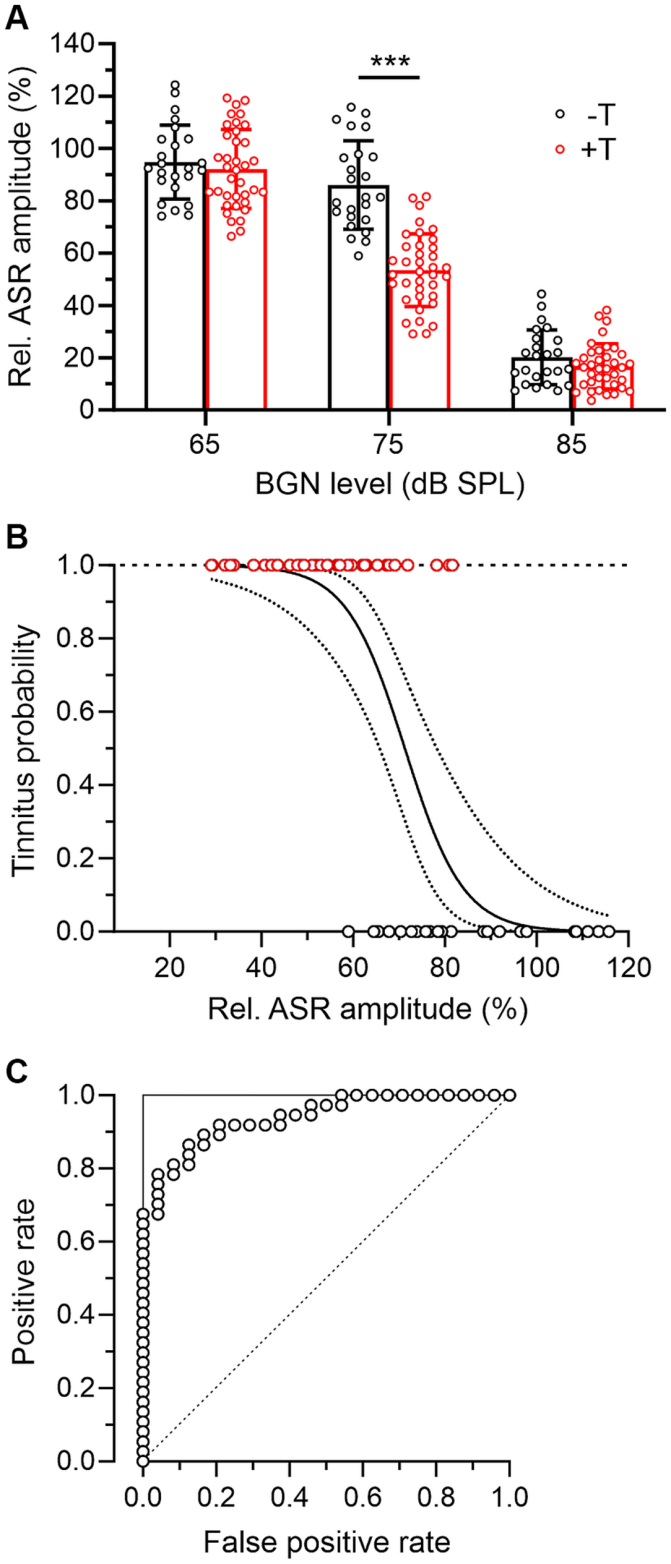
Mice more susceptible to tinnitus induction show increased ASR inhibition in the presence of BGN. **(A)** The bar graph summarizes the inhibitory effect of BGN at three levels on ASR in −T and +T mice. Relative ASR was quantified as the ratio between ASR amplitudes in the presence and absence of BGN x 100%. Note that in the presence of BGN at 65 and 85 dB, inhibition was similar in both groups of mice, whereas BGN at 75 dB suppressed ASR significantly more in +T than in −T mice (two-way ANOVA with SMCT). **(B)** Logistic regression analysis of the probability of tinnitus in mice according to their relative ASR in the presence of 75 dB BGN. The solid line indicates the logistic function modeling the association between a binary outcome (presence of tinnitus in +T mice, red symbols, or absence of tinnitus in −T mice, black symbols) based on a continuous variable (relative ASR amplitude). The dotted lines show the 95% confidence interval for the logistic regression curve. The logistic regression was statistically significant (b_0_ = 10.57 ± 2.74, b_1_ = −0.148 ± 0.039, *p* < 0.001, likelihood ratio test), indicating that the sigmoidal function described the observed data significantly better than the constant function. **(C)** ROC curve (open symbols) reflecting the predictive ability of the logistic regression shown in B. To construct the ROC curve, for each point on the logistic curve that was taken as a threshold for prediction, the number of correctly predicted positive outcomes was plotted against the number of incorrectly predicted positive outcomes (i.e., outcomes predicted as positive that are in fact negative). Note that the shape of the ROC curve is close to the shape of the ideal classifier, represented by the two lines forming a right angle in the upper left corner (solid line). For comparison, the dashed diagonal line shows the ROC curve of a classifier that has no better predictive ability than chance.

Next, we assessed hearing function in all mice based on behavioral measures. As noted above, the +T and −T groups showed similar pre-exposure GPIAS values, and our measurements also showed that pre-exposure raw ASR amplitudes in the absence of BGN did not differ between these groups (−T vs. + T: 0.764 ± 0.222 V vs. 0.847 ± 0.272 V, *p* = 0.269, two-way RM ANOVA with SMCT, *n* = 24 vs. 37). Consistent with previous reports (Gerrard and Ison, 1990), we further observed inhibition of ASR in the presence of BGN, with the degree of inhibition dependent on BGN level (Figure 3A). Interestingly, while ASR inhibition in +T and −T mice was similarly low or high at BGN levels of 65 dB or 85 dB SPL, respectively, at 75 dB SPL BGN elicited significantly greater ASR inhibition, resulting in lower raw ASR amplitudes (ASR_BGN_) in +T mice than in −T mice (−T vs. + T: 0.642 ± 0.187 V vs. 0.445 ± 0.156 V, *p* < 0.01, two-way RM ANOVA with SMCT, *n* = 24 vs. 37). Accordingly, pre-exposure relative ASR amplitudes in the presence of 75 dB BGN were significantly lower in +T than in −T mice (Figure 3A) and significantly correlated with post-exposure GPIAS values in all mice (Spearman’s *r* = 0.641, *p* < 0.001, *n* = 61). In contrast, the correlation between GPIAS and ASR inhibition by BGN at 65 dB or 85 dB was not significant (Spearman’s *r* = 0.048 or 0.091, *p* = 0.714 or 0.488, *n* = 61). We add that relative ASR amplitudes at 75 dB BGN were found to be similar in males and females before noise exposure (*p* = 0.781, unpaired Student’s *t*-test, *n* = 27 and 34). After exposure, ASR inhibition in the presence of 75 dB BGN decreased in −T mice from 86.0 ± 16.9% to 65.3 ± 51.2% while it remained unchanged in +T mice (*p* = 0.004 vs. 0.927, mixed-effects model with Tukey’s multiple comparisons test) (not shown). However, the difference between +T and −T groups remained significant after exposure (*p* = 0.003, two-way ANOVA with SMCT). Thus, the two groups of mice showed differential ASR sensitivity to background sounds. The results also suggest that measuring ASR inhibition in the presence of BGN could represent a simple test of animal susceptibility to noise-induced tinnitus.

When using such a test, it would be essential to know the probability of tinnitus in individual animals with a given BGN-induced ASR inhibition. Therefore, we further investigated the association between the degree of ASR suppression and the presence of tinnitus in mice using regression analysis. Figure 3B shows a logistic regression curve that models the probability of tinnitus in +T and −T mice as a function of a continuous variable, the predictor, which is the relative amplitude of ASR in the presence of BGN. Based on this analysis, mice in which BGN at 75 dB suppressed ASR amplitudes to less than 60% of baseline could be expected to have tinnitus with a probability greater than 80% after acoustic trauma. To estimate the predictive ability of the logistic regression, the receiver operating characteristic (ROC) curve was constructed (Figure 3C). The data suggest that prediction of the presence of tinnitus based on ASR suppression provides highly reliable results because the shape of the ROC curve approximates right-angle lines and the area under the curve (AUC) is equal to 0.94. In comparison, the AUC of an ideal classifier is equal to 1 and a prediction equal to chance has an AUC of 0.5 (Figure 3C).

### Changes in auditory brainstem responses of exposed mice

**Figure 4:**
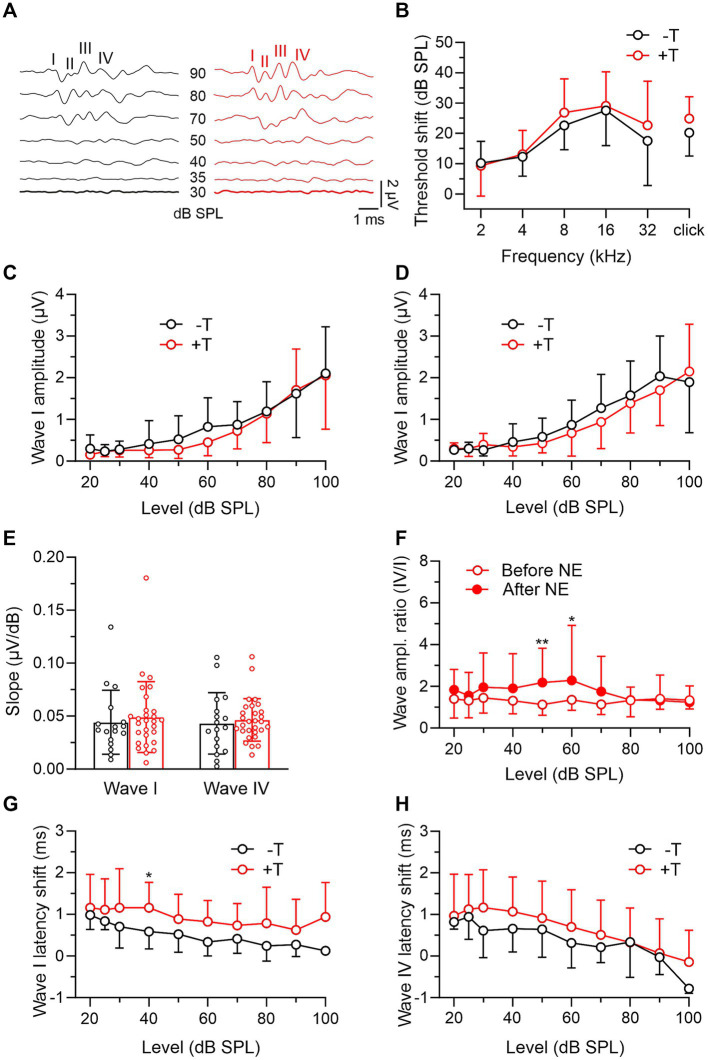
Comparison of post-exposure ABRs in −T and +T mice. **(A)** Representative ABR recordings elicited by clicks of different intensities in mice from the −T and +T group after NE. **(B)** Average increase in thresholds of ABRs to tones and clicks in −T and +T mice. For both groups, the threshold shift was significant at all frequencies of the sound stimuli (*p* < 0.001; −T: mixed-effects model with SMCT, *n* = 24; +T: two-way RM ANOVA with SMCT, *n* = 34). Differences in the shift between −T and +T mice were not significant at any frequency (two-way ANOVA with SMCT). **(C,D)** Amplitude-level functions for click-evoked ABR waves I and IV from the −T and +T groups, respectively. Acoustic trauma led to a significant reduction in amplitudes to values that were not significantly different in +T and −T mice (two-way ANOVA with SMCT). **(E)** Similar slope of ABR growth functions in −T (black) and +T (red) mice after NE (*p* = 0.820 and 0.915 for waves I and IV, respectively; two-way ANOVA with SMCT). **(F)** Increased ratio of wave IV and I amplitudes after NE in +T mice (*p* = 0.004, mixed-effects model; **p < 0.01, *p < 0.05, SMCT, *n* = 29). **(G,H)** Plots show prolongation of ABR wave I and IV latency in −T and +T mice after NE. The changes were greater in +T mice, so that the latency vs. stimulus intensity curves were significantly different in the mouse groups after exposure (*p* < 0.001 and *p* = 0.014, two-way ANOVA; *p < 0.05, SMCT).

In the last part of the experiments, we compared auditory function in +T and −T mice after noise exposure. Both groups showed similar increases in ABR thresholds of about 10 to 30 dB, with the largest shift observed in the 8–16 kHz band, which roughly corresponds to the frequency of narrowband noise used for traumatic overexposure (Figures 4A,B). Furthermore, we observed a decrease in the maximum amplitude of both early and late ABR waves by more than half (*p* < 0.001, mixed-effects model with SMCT) and a corresponding decrease in the slope of the input–output functions (*p* < 0.001 for wave I in both mouse groups and *p* < 0.01 or *p* < 0.001 for wave IV in −T or +T mice, respectively; mixed-effects model with SMCT) to values that were not significantly different in +T and −T mice (Figures 4C–E). The IV/I ratio increased after exposure for ABRs elicited by moderate-intensity stimulation in +T mice (Figure 4F), whereas it did not change in −T mice across the entire range of stimulation levels (mixed-effects model with SMCT; not shown). The latencies of wave I and wave IV peaks were prolonged after exposure in both mouse groups (*p* < 0.001, mixed-effects model), with the change significantly greater in +T mice (Figures 4G,H). These observations indicate noise-induced hearing loss caused by acoustic trauma in both groups of mice, and the differences between +T and −T mice are consistent with previously published audiometric correlates of noise-induced tinnitus.

## Discussion

The results presented in this study point to increased ASR inhibition in the presence of BGN as a possible behavioral correlate of susceptibility to noise-induced tinnitus. We used BGN at three levels, of which only 75 dB had a differential effect on ASR in −T and +T mice (Figure 3A). The remaining two levels resulted in either insignificant modulation (65 dB) or strong suppression (~80% at 85 dB) of ASR, similarly for both groups of mice. Earlier work detailing the dependence of rat ASR on BGN intensity found an inverted-U-shaped biphasic curve showing an initial increase in response at levels up to the optimal value (~75 dB) and a subsequent decrease at levels above this value (Ison and Silverstein, 1978). Further studies have shown that in the case of narrowband noise dominated by high-frequency components, the dependence curve lacks a potentiation phase and increasing noise intensity only suppresses ASR (Gerrard and Ison, 1990). Thus, our data obtained using 10 kHz centered narrow-band noise support that the different ASRs in −T and +T mice were not due to differential potentiation, but to a stronger inhibition of ASR in the +T group. The mechanisms behind these differences were not further investigated in this study. We do not assume that they simply reflect possible differences in sensory masking in +T and −T mice. This mechanism depends on the spectral characteristics of the masker and the signal, with the most effective masking expected when their predominant frequencies are similar (Gerrard and Ison, 1990). In our experiments, white noise, whose higher frequencies could be sensitive to masking by 75 dB BGN centered at 10 kHz, was used as the eliciting stimulus, and the similarity between the ABR audiograms of +T and −T mice does not suggest differences in their reactivity to sounds in this frequency range. On the other hand, the increased ASR suppression in +T mice may reflect weaker inhibitory GABAergic signaling, which has been previously proposed to accompany reduced global neuronal activity in gerbils susceptible to noise-induced tinnitus (Ahlf et al., 2012; Tziridis et al., 2015). The increased slope of growth functions for ABR amplitudes that we observed in +T mice would be consistent with this assumption (Koebis et al., 2019; but see Tziridis et al., 2015 for differences). Thus, intrinsic variability in the strength of GABAergic inhibition in the auditory pathway of mice could underlie the differential ability of their auditory circuits to adapt to prolonged background noise, and hence altered sensorimotor gating in +T mice (Ison and Silverstein, 1978; Swerdlow et al., 2000; Resnik and Polley, 2021; Burghard et al., 2022).

The classification of mice into groups differing in susceptibility to noise-induced tinnitus critically depends on the detection of phantom perception after acoustic trauma. For this purpose, we used the GPIAS paradigm (Turner et al., 2006), as have previous similar studies (Ahlf et al., 2012; Li et al., 2015; Miyakawa et al., 2019). This test has its limits and depends on parameters such as the magnitude of ASR and the severity of hearing loss after noise exposure (reviewed in Eggermont and Roberts, 2015; Galazyuk and Hebert, 2015; Shore et al., 2016; Henton and Tzounopoulos, 2021). Our data show that +T and −T mice had similar pre-exposure GPIAS values and that for all mice in this study, these values were not significantly correlated before and after exposure. This indicates that the assignment of mice to one of the groups was not preset by their basal inhibition of the startle reflex by gap pre-pulse. Furthermore, after exposure, the +T and −T groups showed similar raw ASR amplitudes in the presence of 75 dB BGN, and GPIAS values were not significantly correlated with the relative ASR amplitudes at BGN. This indicated that the failure of the GPIAS in +T mice after trauma was not due to excessive suppression of the ASR by the BGN, which might otherwise lead to a “floor effect,” reducing the capacity of the startle reflex for further inhibition by the gap pre-pulse in the GPIAS test (Galazyuk and Hebert, 2015). Thus, the data supports that the differentiation of +T and −T mice based on their postexposure GPIAS indeed reflects their differential sensitivity to tinnitus induction. This assumption is further supported by the fact that after exposure, +T mice exhibit neurophysiological correlates of tinnitus such as an increase in the ratio between the amplitudes of ABR waves IV and I and a more pronounced prolongation of the latency of these waves (Mohrle et al., 2019).

In conclusion, measurement of ASR inhibition by BGN appears to be a simple non-invasive method of predicting the development of noise-induced tinnitus in laboratory rodents. If future experiments demonstrate similar differential modulation of the acoustic startle eyeblink response in humans, an analogue test could help identify individuals at risk of tinnitus and introduce proactive use of hearing protection and medication.

## Data availability statement

The raw data supporting the conclusions of this article will be made available by the authors, without undue reservation.

## Ethics statement

The animal study was approved by the Ethics Committee of the Institute of Experimental Medicine, Academy of Sciences of the Czech Republic. The study was conducted in accordance with the local legislation and institutional requirements.

## Author contributions

NR: Data curation, Investigation, Methodology, Writing – review & editing, Conceptualization, Validation. ŠS: Data curation, Investigation, Methodology, Writing – review & editing. ZB: Data curation, Investigation, Methodology, Software, Writing – review & editing. NJ: Investigation, Writing – review & editing. AM: Investigation, Writing – review & editing. OP: Investigation, Writing – review & editing. RT: Conceptualization, Funding acquisition, Methodology, Project administration, Resources, Supervision, Writing – original draft, Writing – review & editing.
